# Elevated risk of infection with SARS-CoV-2 Beta, Gamma, and Delta variant compared to Alpha variant in vaccinated individuals

**DOI:** 10.1126/scitranslmed.abn4338

**Published:** 2022-07-21

**Authors:** Stijn P. Andeweg, Harry Vennema, Irene Veldhuijzen, Naomi Smorenburg, Dennis Schmitz, Florian Zwagemaker, Arianne B. van Gageldonk-Lafeber, Susan J. M. Hahné, Chantal Reusken, Mirjam J. Knol, Dirk Eggink, SeqNeth Molecular surveillance group, RIVM COVID-19 Molecular epidemiology group

**Affiliations:** ^1^ Centre for Infectious Disease Control, National Institute for Public Health and the Environment (RIVM), Antonie van Leeuwenhoeklaan 9, 3720 BA, Bilthoven, Netherlands.

## Abstract

The extent to which severe acute respiratory syndrome coronavirus 2 (SARS-CoV-2) variants of concern (VOC) break through infection- or vaccine-induced immunity is not well understood. We analyzed 28,578 sequenced SARS-CoV-2 samples from individuals with known immune status obtained through national community testing in the Netherlands from March to August 2021. We found evidence of an increased risk of infection by the Beta (B.1.351), Gamma (P.1), or Delta (B.1.617.2) variants compared to the Alpha (B.1.1.7) variant after vaccination. No clear differences were found between vaccines. However, the effect was larger in the first 14-59 days after complete vaccination compared to ≥60 days. In contrast to vaccine-induced immunity, there was no increased risk for re-infection with Beta, Gamma or Delta variants relative to Alpha variant in individuals with infection-induced immunity.

## Teaser

SARS-CoV-2 genomic epidemiology shows increased breakthrough but not re-infection rates, by the Beta, Gamma, or Delta variants compared to Alpha.

## INTRODUCTION

The worldwide spread of severe acute respiratory syndrome coronavirus 2 (SARS-CoV-2) has been associated with the evolution and emergence of mutated viral variants. Although many nucleotide mutations are synonymous and do not directly impact viral fitness, multiple amino acid substitutions in functional domains of the Spike protein have been observed, some of which have been shown to impact transmissibility, disease severity, and pre-existing immunity (*1*).

SARS-CoV-2 variants with multiple mutations that are suspected to impact viral virulence, transmission, or efficacy of diagnostics, vaccines and antivirals have been designated variants of concern (VOC) (*2*). As of December 2021, five VOCs had been defined by the ECDC and WHO: Alpha (B.1.1.7, first detected in September 2020 in the United Kingdom), Beta (B.1.351, first detected in May 2020 in South Africa), Gamma (P.1, first detected in November 2020 in Brazil), Delta (B.1.617.2, first detected in October 2020 in India) and Omicron (B.1.1.529, first detected in November 2021 in multiple countries) (*2*). All these VOCs contain amino acid substitutions in the receptor binding domain (RBD) and N-terminal domain (NTD) of the Spike protein, which are known to be the main targets of neutralizing antibodies. Several studies have shown decreased neutralization of VOCs by convalescent and post-vaccination sera in vitro, with little or no reduction in sensitivity for the Alpha variant, and the highest reduction in sensitivity of Beta and Omicron, and to a lesser extent, of Gamma and Delta *(* *3* *–* *6* *)*.

These observations, and the rapid global spread of VOC like Alpha and then Delta, caused concerns in early 2021 that SARS-CoV-2 VOCs may escape pre-existing immunity and may still be able to infect and be transmitted by vaccinated and previously infected individuals. There are indications that the vaccine effectiveness (VE), especially against SARS-CoV-2 infection or mild COVID-19, is lower for the Beta, Gamma and Delta variant*(* *7* *)*. After vaccination, an increase in the proportion of individuals infected with the Alpha and Beta variants compared to the parental strain was observed (*8*). Less is known about the association between the Beta and Gamma variants and re-infection. In a (case-)matched test-negative design, no differences were found for infection-induced protection between the Alpha and Delta variant (*9*). Although an ecological study from the UK did not find an increase in the re-infection rate for the Alpha variant relative to pre-existing variants in the last quarter of 2020 (*10*), increased risk of re-infection by the Beta, Gamma, or Delta variants compared to the Alpha variant still needs to be established. For the Omicron BA.1 variant we showed that infection- and vaccine-induced protection was largely reduced compared with Delta (*11*).

In January 2021, the COVID-19 vaccination program was rolled out in the Netherlands, which first prioritized health care workers, nursing home residents, and the elderly. Current approved vaccines are either based on an mRNA vector (Comirnaty (BNT162b2, BioNTech/Pfizer, Spikevax (mRNA-1273/Moderna) or on an adenovirus-based vector system (Vaxzevria (ChAdOx1/AstraZeneca), Ad26.COV2.S (Janssen)) and are aimed at eliciting a Spike protein-specific humoral immune response that induces antibodies that prevent virus entry and replication (*12*, *13*). All persons 12 years and older have been offered COVID-19 vaccination in the Netherlands as of July 2021. As of May 2022, 86% of all adults were fully vaccinated, and 89% received at least one dose (*14*). In the vaccination program in the Netherlands, Comirnaty has been the most commonly used vaccine and has been offered to all age groups (76.0% of all administered doses). Spikevax has been used primarily for residents of long term care facilities, health care workers, high medical risk groups, and later for the general population <60 years old (8.5% of all administered doses). Vaxzevria has been used mostly in health care workers and the 60-65 year old age group (12.1% of all administered doses). Janssen COVID-19 vaccine has been used mostly in the 50-59 year old age group and young adults (3.4% of all administered doses) (*15*). Vaccination has proven to be highly effective against COVID-19, especially against hospitalization and death, and has reduced the secondary attack rate within households (*16*–*20*).

Infection with SARS-CoV-2 can also elicit a protective immune response but re-infections are possible. Studies comparing infection rates during the first and second surge of the SARS-CoV-2 pandemic between people who tested RT-PCR or antigen negative and positive in Denmark, Austria and Italy reported protection against repeat infection of 81%, 91% and 94%, respectively (*21*–*23*). A prospective cohort study among health care workers in the UK found a 84% lower risk of infection after a previous infection (*24*).

In the Netherlands, randomly selected SARS-CoV-2 RT-PCR positive specimens are sequenced to continuously monitor changes in the virus (*25*). The Alpha variant started to increase rapidly from January 2021 and quickly became the dominant strain in the Netherlands. From June 2021, the Delta variant increased rapidly and caused nearly all infections from August 2021 onwards. In this study we aimed to investigate whether vaccine- or infection-induced immunity protected less well against infection by specific variants using national epidemiological and molecular surveillance data from March to August 2021. We employed a case-only approach in which we compared the immune status among cases infected with the Beta, Gamma, or Delta variants versus the Alpha variant. We assessed the relative effectiveness of vaccination against Beta, Gamma or Delta compared to Alpha variant (*26*). Similarly, we analyzed the protective effect of previous SARS-CoV-2 infection against a new infection with Beta, Gamma or Delta vs Alpha variants. Previous studies used a similar design and found relative protection differences between Alpha-Beta and Delta-Omicron BA.1 (*8*, *11*).

## RESULTS

From 1 March to 31 August 2021, a total of 661,658 SARS-CoV-2 positive cases were collected in the national surveillance database (Table 1). Of these, 38,261 (5.8%) cases were partially vaccinated individuals, 25,933 (3.9%) were fully vaccinated individuals, and 10,565 (1.6%) had a known previous infection (Fig. S1). Among vaccinated individuals, most received Comirnaty (65.0%), followed by Vaxzevria (19.3%), Janssen COVID-19 vaccine (9.8%) and Spikevax (5.9%). We included data of 29,305 samples that were sequenced through the national SARS-CoV-2 surveillance program (Table 1). In addition, 1,516 additional randomly selected samples were sequenced to gain insight into variants present during infections after vaccination and re-infections.

**Table 1.** Characteristics of notified SARS-CoV-2 positive cases overall and for which variant information was available, 1 March to 31 August 2021, the Netherlands.

	**Notifications**	**Variant information from genomic surveillance**	**Variant information from additional sampling**
Total	661,658	29,305	1,516
Immune status			
Naïve	487,063 (73.6%)	20,804 (71.0%)	NA
Recently vaccinated	47,565 (7.2%)	2,140 (7.3%)	18 (1.2%)
Partially vaccinated	38,261 (5.8%)	2,016 (6.9%)	707 (46.6%)
Fully vaccinated	25,933 (3.9%)	1,791 (6.1%)	516 (34.0%)
Previous infection	10,565 (1.6%)	284 (1.0%)	191 (12.6%)
Vaccinated and previous infection	2,065 (0.3%)	62 (0.2%)	49(3.2%)
Unknown	50,206 (7.6%)	2,208 (7.5%)	35 (2.3%)
Age group			
0-9	42,666 (6.4%)	1,818 (6.2%)	4(0.3%)
10-19	125,782 (19.0%)	5,869 (20.0%)	111 (7.3%)
20-29	157,896 (23.9%)	7,018 (23.9%)	283 (18.7%)
30-39	92,400 (14.0%)	4,162 (14.2%)	187 (12.3%)
40-49	85,492 (12.9%)	3,851 (13.1%)	222 (14.6%)
50-59	87,112 (13.2%)	3,652 (12.5%)	265 (17.5%)
60-69	44,226 (6.7%)	1,828 (6.2%)	251 (16.6%)
70-79	21,074 (3.2%)	848 (2.9%)	86 (5.7%)
80+	5,010 (0.8%)	259 (0.9%)	107 (7.1%)
Sex			
Male	330,247 (49.9%)	14,437 (49.3%)	629 (41.5%)
Female	331411 (50.1%)	14,868 (50.7%)	692 (58.5%)
Symptoms			
Yes	556,214 (84.1%)	25,478 (86.9%)	1355 (89.4%)
No	66,593 (10.1%)	2,248 (7.7%)	121 (8.0%)
Unknown	38,851 (5.9%)	1,579 (5.4%)	40 (2.6%)
Month (sampling date)			
March	149,103 (22.5%)	5,408 (18.5%)	177 (11.7%)
April	171,534 (25.9%)	4,621 (15.8%)	335 (22.1%)
May	114,536 (17.3%)	4,874 (16.6%)	137 (9.1%)
June	24,904 (3.8%)	3,162 (10.8%)	97 (6.4%)
July	146,978 (22.2%)	6,620 (22.6%	438 (28.9%)
August	54,603 (8.3%)	4,620 (15.8%)	331(21.8%)

**Fig. 1.**
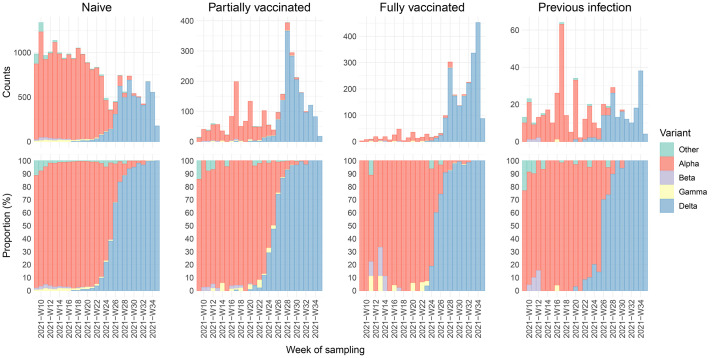
Variants found in SARS-CoV-2 positive samples of individuals with naïve (unvaccinated and no known previous infection), vaccine-induced, or infection-induced immune status. Number of naïve, partially vaccinated, fully vaccinated, and reinfected documented SARS-CoV-2 positive individuals by variant from March 1 to August 31, 2021 (upper panels) and proportion of the respective groups (lower panels) per week of sampling (in ISO 8601 format).

Up until June 2021, 94.4% (14,068 of 14,903) of infections were caused by the Alpha variant, with a small proportion caused by the Beta (1.3%) and Gamma (1.3%) variants. The proportion of Delta increased from 0.9% (42 of 4874) in May to 98.7% (4561 of 4620) in August 2021. This pattern was observed over different immune statuses (Fig. 1, Fig. S2). In total, 17,890 (58.0%) Alpha, 209 (0.7%) Beta, 250 (0.8%) Gamma, 11,937 (38.7%) Delta and 535 (1.7%) other variant sequences were observed.

**Fig. 2.**
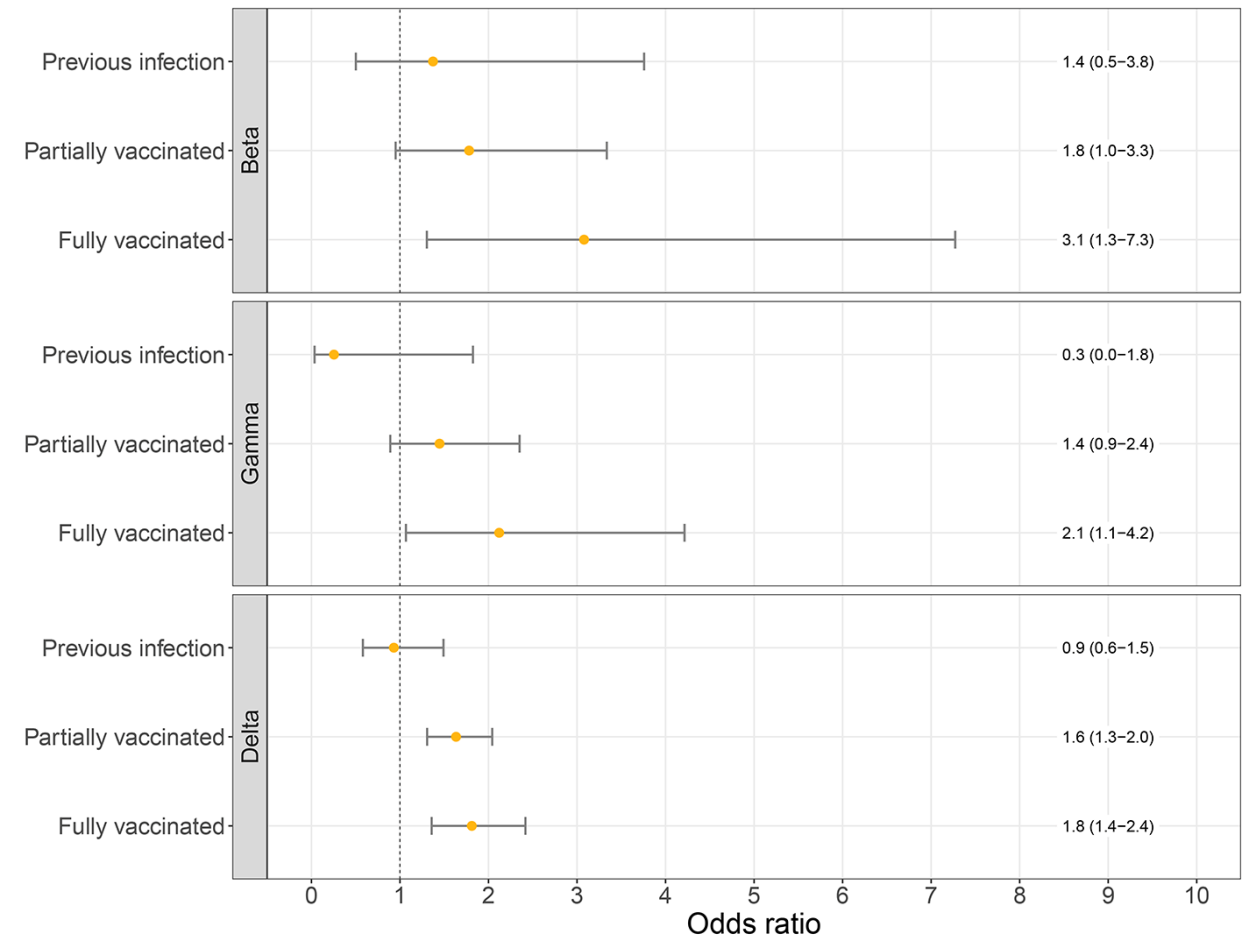
Odds ratios of the logistic regression models for the association between immune status and VOC (Beta, Gamma or Delta over the Alpha variant). Logistic regression models are adjusted for week of sampling, sex and 10-year age group. Error bars correspond to the 95% confidence intervals.

Logistic regression analysis showed that full vaccination was significantly associated with infection by the Beta, Gamma or Delta variants compared to the Alpha variant (adjusted OR: 3.1 (95% CI: 1.3-7.3); 2.1 (95% CI: 1.1-4.2); 1.8 (95% CI: 1.4-2.4); respectively; Fig. 2). The association for partial vaccination was less strong and not significant for Beta and Gamma but was significant for Delta when compared to Alpha (adjusted OR: 1.6 (95% CI: 1.3-2.0); Fig. 2). We did not find a significant association between previous infection and the Beta, Gamma or Delta variant over Alpha (adjusted OR: 1.4 (95% CI 0.5-3.8); 0.3 (95%CI 0.0-1.8; 0.9 (95%CI 0.6-1.5), respectively; Fig. 2). Younger age was significantly associated with prevalence of the Delta variant, age groups 10-19 (adjusted OR: 1.4 (95% CI: 1.1-1.8)) and 20-29 (adjusted OR: 1.3 (95% CI: 1.0-1.7)) years old, which highlights the importance of adjustment for age group (Fig. S3 shows multivariable analysis). Analysis of data using only genomic surveillance, and excluding data from additional sampling of vaccinated and re-infected cases, revealed similar odds ratios for Delta variant (OR: 1.7 (95% CI 1.2-2.3) for fully vaccinated), although no significance was measured for Beta and Gamma likely due to insufficient observations for these variants.

When stratified by vaccine type, the point estimates differ somewhat between the different vaccines although the confidence intervals are wide and overlapping (Table 2). The association between partial vaccination and the Delta variant was significant for Comirnaty (OR: 1.7 (95% CI 1.3-2.1) and Vaxzevria (OR: 2.0 (95% CI 1.2-3.4) but not Spikevax (OR: 1.0 (95% CI 0.5-1.7). In addition, we stratified the fully vaccinated by time since vaccination. The association for individuals with less time (14-59 days) between onset and last dose was higher (OR: 2.3 (95%CI 1.6-3.4)) compared to individuals with ≥60 days (OR: 1.4 (95%CI 0.9-2.0)) for the Delta variant. A similar trend was observed for the Beta variant and Gamma variant, although these analyses resulted in wider confidence intervals (Table 2).

**Table 2.** Odds ratios (ORs) and 95% confidence intervals (CIs) for the association between immune status and VOC (Beta, Gamma or Delta over the Alpha variant) by vaccine type and days between onset and last dose, both adjusted for week of sampling, sex and 10-year age group.

	**Beta** **OR (95% CI)**	**Gamma** **OR (95% CI)**	**Delta** **OR (95% CI)**
Naïve	Reference	Reference	Reference
Partially vaccinated			
Comirnaty	1.2 (0.3-4.4)	2.0 (1.0-3.9)	1.7 (1.3-2.1)
Spikevax	n/a	2.7 (0.6-11.3)	1.0 (0.5-1.7)
Vaxzevria	2.0 (1.0-4.0)	1.0 (0.5-2.1)	2.0 (1.2-3.4)
Fully vaccinated			
Comirnaty	3.2 (1.4-7.6)	2.1 (1.0-4.7)	2.1 (1.4-3.2)
Spikevax	n/a	n/a	1.3 (0.4-4.1)
Vaxzevria	n/a	2.7 (0.6-12.0)	1.4 (0.9-2.3)
Janssen	n/a	3.7 (0.5-31.0)	2.2 (1.1-4.0)
Naïve	Reference	Reference	Reference
Fully vaccinated			
14-60 days	3.4 (1.3-8.8)	2.9 (1.2-6.8)	2.3 (1.6-3.4)
>60 days	2.1 (0.3-15.4)	1.5 (0.5-4.2)	1.4 (0.9-2.0)

## DISCUSSION

Using national epidemiological and whole genome sequencing surveillance data from March to August 2021 in the Netherlands, our analysis provides evidence for an increased risk of infection by the Beta, Gamma, or Delta variants compared to the Alpha variant after full vaccination, regardless of the vaccine used. This indicates lower vaccine effectiveness against infection with the Beta, Gamma and Delta variants compared to the Alpha variant. No clear differences between vaccine types were observed as confidence intervals largely overlapped. Interestingly, we did not find a significant difference between susceptibility to any of the investigated VOCs among individuals with immunity due to a previous infection compared to naïve individuals. Of note, these analyses do not aim to determine the probability of getting infected after vaccination or previous infection, but rather calculate the likelihood of getting infected with specific VOCs.

The association with vaccination status was higher for Beta and Gamma (OR of 3.1 and 2.1, respectively) than for Delta (OR of 1.8), although confidence intervals for Beta and Gamma were wide because of low numbers. This is in line with literature showing lower vaccine effectiveness estimates against infection for Beta and Gamma compared to Delta (*7*). An OR for Delta of 1.8 implicates a reduction of vaccine effectiveness from ~90% to 80%, which has been shown in the UK (*27*, *28*). Current literature still shows high vaccine effectiveness of 90-95% against severe COVID-19 for the Delta variant (*7*, *19*), which is reassuring. However, note that with very high vaccine effectiveness, a difference of a factor 1.5-2.0 between two variants could go unnoticed, as it would only mean a decrease of effectiveness of for example 95 to 92%. Nonetheless, comparisons of clinical trials for COVID-19 vaccines variant must consider these differences.

Spike binding and neutralization have been shown to be substantially reduced against Beta, Gamma, and Delta, with the largest reduction in neutralization against Beta (*3*–*5*), which is consistent with our results. This observation did not differ for infection- or vaccine-induced immunity, although convalescent sera from mild infections showed lower levels of neutralization potency to VOCs compared to hospitalized cases and vaccinated individuals (*3*). However, in Alpha and Beta a reduction was not observed for T-cell-mediated immunity (*29*).

We observed a larger effect of vaccination in the first 14-59 days after vaccination (i.e., OR 2.3 (95%CI 1.6-3.4) for Delta) compared to 60 days and longer (i.e., OR 1.4 (95%CI 0.9-2.0) for Delta), suggesting that the difference in VE between Delta and Alpha variant reduces over time since vaccination, possibly due to waning immunity. The decline of VE with time since vaccination is described in a systematic review (*30*). A large cohort study describes an effect of waning and a small effect of the circulating variant (i.e., Delta vs non-Delta) on the VE against SARS-CoV-2 infection (*31*). They observed a non-delta VE of 97% and a delta VE of 93% one month after vaccination, which meant a ratio of 2.3 between non-delta VE and delta VE. Four to five months post vaccination, VE estimates of 67% and 53% for non-delta and delta were observed respectively, at a ratio of 1.4, and this corresponds with our results. Given the broad and sometimes overlapping confidence intervals of these data, however, the differences need to be interpreted with caution.

We found no association between previous infection and a new infection with Beta, Gamma, or Delta versus Alpha, suggesting that there is no difference in protection from a previous infection between Beta, Gamma, or Delta variants compared to the Alpha variant. This is in line with the similar relative risk reductions for re-infection found for the Alpha and Delta variant (*9*). Early studies showed that previous infection conferred better protection than vaccination without previous infection during the Delta period (*32*, *33*). Best protection was induced by the combination of vaccination and previous infection. However, primary infection comes with a risk of hospitalization or death, especially in older persons or individuals with underlying conditions. Even if infection-induced immunity protects better against re-infection with novel variants, vaccination is preferred over infection to protect individuals against severe disease as the cumulative risk from two infections should be considered.

There are some limitations to our study, including the issue of asymptomatic or mild cases with low viral load being less likely to identify, and only detectable infections could be sequenced and included in analyses. In addition, sequencing is more successful in samples with low to medium Ct values (high to medium viral load). If infection with Beta, Gamma or Delta leads to lower Ct values than Alpha, and Ct values are higher for infections after vaccination (*34*–*36*), this could have led to an overestimation of the studied association. Another limitation is that prior infections could go undetected, especially if it occurred during the first wave when there was no mass scale testing capacity. This could lead to an underestimation of cases with a previous infection, as we do not directly measure pre-existing infection-induced immunity.

In conclusion, our results confirm a lower vaccine effectiveness against infection for the Delta variant, and similarly the Beta and Gamma variant, compared to Alpha. This effect was largest early after complete vaccination. These findings are informative for considerations on vaccine updates, future vaccination and pandemic control strategies and similar analyses for novel variants, such as Omicron variants or other future variants.

## MATERIALS AND METHODS

### Study design

The aim of this study was to assess if there is an increased risk of infection for the Beta, Gamma, or Delta variant compared to Alpha for individuals with infection- or vaccine-induced immunity. Start and end points for used SARS-CoV-2 isolates were based on the variant circulation measured in the Dutch national SARS-CoV-2 molecular surveillance program (*37*). The starting point was defined by the dominance of the Alpha variant as this is our dependent variable in the regression analysis and endpoint was based on the disappearance of this variant from the surveillance data, as almost all isolates contained Delta variant from Augustus up to November 2021.

### Data

Persons testing positive for SARS-CoV-2 either by community testing or in a hospital are notified by Public Health Services (PHS) to the national surveillance database. Community testing is available through the PHS. Testing is encouraged for individuals experiencing COVID-19-like symptoms, contact with a positive case, returning from another country, or upon a positive self-test. Data relevant for source and contact tracing and for surveillance was collected in the national surveillance database through a telephone interview, including data on vaccination status (i.e., number of doses, type of vaccine, and date of vaccination).

The Dutch national SARS-CoV-2 molecular surveillance program sequences whole virus genomes of randomly selected SARS-CoV-2 positive specimens from both community testing (via PHS) and hospitals, using nationwide geographical distribution. In the current analysis, only samples with information on vaccination status or previous infection can be used. This information is collected in the national surveillance database and linked to sequence data using a sample identifier supplied during community testing. Sequences from hospital samples (5,893 out of the total 42,662 (13.8%) sequences of the SARS-CoV-2 genomic surveillance samples) and 7,464 of the 36,769 sequenced community samples were excluded as these could not be linked to the national surveillance database for required meta-data. As our study period was during the roll out of the vaccination program, the number of sequenced samples among vaccinated persons was small. Therefore, additional sequencing was done on a random sample of positive tests of vaccinated persons to increase the statistical power of the study for analysis of the association between vaccination and variant, and this was also done for positive tests from persons with a previous infection. This additional random sampling was done on a triweekly basis and resulted in an additional inclusion of 1,516 cases. In the current analyses, cases with a sampling date between March 1 and August 31, 2021, were included.

### Ethics

The Centre for Clinical Expertise at the National Institute for Public Health and the Environment (RIVM) assessed the research proposal following the specific conditions as stated in the law for medical research involving human subjects. The work described was exempted for further approval by the ethical research committee. Pathogen surveillance is a legal task of the RIVM and is carried out under the responsibility of the Dutch Minister of Health, Welfare and Sports. The Public Health Act provides that RIVM may receive pseudonymized data for this task without individual consent.

### RT-PCR amplification and Nanopore sequencing

Most isolates were sequenced according to the following representative sequence method. Total nucleic acid from combined nasopharyngeal and oropharyngeal swabs were extracted using MagNApure 96 (MP96) with the total nucleic acid kit small volume (Roche). Total nucleic acid was eluted in 50 μl Tris EDTA buffer. SARS-CoV-2 specific RT-PCR amplification and sequencing was performed using the Nanopore protocol based on the ARTIC v3 amplicon sequencing protocol (*38*). Several modifications to the protocol were made for optimization: 1) The total volume of the cDNA reaction is 12μl with a volume of 0.4μl Superscript IV instead of 0.6μl. 2) primer concentrations and primer sequence were adjusted for several amplicons to optimized amplicon yield and to match novel variants. Updated primer sequences are available upon request. 3) No distinction was made on the basis of Cp value, PCR was performed using 47 cycles. After the combination of PCR reactions A and B, the samples were quantified with the Qubit, samples with a concentration >35ng/μl were diluted to 6ng/μl in water. 5 μl of diluted PCR mix was used in the end-prep reaction. This end-prep is incubated for 15 min at 20°C and 15 min at 65°C. Barcoding was performed using the NEBNext Ultra II Ligation Module (E7595). In short, 1.3 μl end-prepped DNA was added to 2.5μl water, 6μl NEBNext Ultra II Ligation Master Mix, 0.2μl NEBNext Ligation Enhancer and 2 μl Native barcode SQK-LSK109 (EXP-NBD196). The Barcoding was incubated for 30 min at 20°C and 20 min at 65°C. Barcoded fragments were washed with twice with 870 μl short fragment buffer (SFB), once with 150 μl ethanol and eluted in 74 μl after 4 min incubation with the beads. Adapter ligation was perfomed using NEBNext Quick Ligation Module (NEB) in a total volume 50 μl using 25 μl of AMPure XP beads. After washing with 125 μl short fragment buffer (SFB), the pellet was resuspended in 15.5 μl elution buffer. Finally, 45ng of library preparation was loaded on a flowcell (Nanopore) and sequencing was performed on a R9.4.1 flow cell multiplexing 48 up to 96 samples per sequence run for a run-time of 30 hours on a GridION (Nanopore).

GridION data was analyzed to get consensus genomes, with the SARS2seq pipeline and additional manual curation (*39*). These genomes were analyzed with Pangolin (version 3.1.11) and NextClade (version 1.3.0) to get a final variant call (*40*, *41*).

### Vaccination and previous infection status

Vaccination status is determined relative to the date used for statistics (DUFS). For symptomatic cases, this is the date of symptom onset or, if missing, the date of a positive test result minus 2 days. For asymptomatic cases, the DUFS is the date of positive test result. Fully vaccinated is defined as having received two doses of Comirnaty, Spikevax or Vaxzevria at least 14 days before DUFS or one dose of Janssen COVID-19 vaccine at least 28 days before DUFS. Partially vaccinated is defined as having received one dose of Comirnaty, Spikevax or Vaxzevria at least 14 days before DUFS, or two doses of Comirnaty, Spikevax or Vaxzevria less than 14 days before DUFS. A case is defined as recently vaccinated after one dose of Comirnaty, Spikevax or Vaxzevria 0-13 days or Janssen COVID-19 vaccine 0-27 days before DUFS. Individuals with a subsequent positive RT-PCR or antigen test result with an interval of at least 8 weeks after a previous positive test, including a period without symptoms, were defined as re-infections. This is either reported in the notification by the PHS or identified using record linkage by date of birth, sex, and 6-digit postal code. Previous infection history was mostly based on a previous positive test in the national surveillance database, although in a small number of cases it was based on a self-reported positive test (13 cases, 2.7% of all previous infections).

### Statistical analyses

We compared the proportion of the four VOCs (Alpha, Beta, Gamma and Delta variant) between four immune status groups: 1) unvaccinated cases without a known previous infection (naïve), 2) partially vaccinated cases without a known previous infection, 3) fully vaccinated cases without a known previous infection, 4) unvaccinated cases with a previous infection. In a secondary analysis, fully vaccinated cases were further stratified by time between infection and last vaccination (<60 days versus >=60 days). Cases who were recently vaccinated, irrespective of their previous infection status, were excluded from the analyses, due to a possible incomplete immune response. Since the number of vaccinated cases with a previous infection was small (*n* = 111) this group was excluded.

The association between immune status and the Beta, Gamma and Delta variant was assessed using multinomial logistic regression. Immune status (group 2: partially vaccinated, group 3: fully vaccinated and group 4: previous infection versus group 1: naïve) was included in the model as the independent variable and Beta, Gamma or Delta vs Alpha as the dependent variable. We estimated odds ratios (ORs) with 95% confidence interval (CI) for any vaccine type and separately for Comirnaty, Spikevax, Vaxzevria and Janssen COVID-19 vaccine. An odds ratio of 1 would mean that the protection from vaccination or previous infection is the same against Beta, Gamma or Delta infection and Alpha infection. An odds ratio of >1 would mean that vaccination or previous infection give lower protection against Beta, Gamma or Delta infection than against Alpha infection. An additional analysis was performed on the time since vaccination, stratifying the fully vaccinated by 14-59 and more than 60 days between complete vaccination and DUFS. As calendar time is both related to vaccination uptake and prevalence of a certain variant, i.e., a confounder, we correct for calendar week of sample date in all regression models. We used a natural cubic spline (5 kn) to adjust for calendar week to not restrict the association between calendar time and variant prevalence to follow a certain form, e.g., linear or exponential. In addition, all analyses were also adjusted for 10-year age group (40-49 years as reference) and sex.

## Supplementary Materials

### Other Supplementary Material for this manuscript includes the following:

MDAR Reproducibility Checklist

## SeqNeth Molecular surveillance group:

Janke Schinkel^2^, Matthijs R.A. Welkers^2^, Marcel Jonges^2^, Jelle Koopsen^2^, Menno D. de Jong^2^, Richard Molenkamp^3^, David F. Nieuwenhuijse^3^, Reina S. Sikkema^3^, Bas B. Oude Munnink^3^, Marion Koopmans^3^, Adri van der Zanden^4^, Laura Manrho^4^, Jessica de Beer^4^, Stefan A. Boers^5^, Erin Meijers^5^, Tom Vreeswijk^5^, Igor A. Sidorov^5^, Djoo Dunk^6^, Pieter W. Smit^6^, Suzan D. Pas^7^, Jaco J. Verweij^7^, Joep J. J. M. Stohr^7^, Jozef Dingemans^8^, Brian van der Veer^8^, Lieke van Alphen^8^, Paul Savelkoul^8^, Hubert G.M. Niesters^9^, Erley F. Lizarazo Forero^9^, Monika A Fliss^9^, Lilli Gard^9^, Anniek A.N. Tanja^10^, Rob Schuurman^10^, Annemarie M.J. Wensing^10^, L. Marije Hofstra^10^, Jordy P.M. Coolen^11^, Janette C. Rahamat-Langendoen^11^, Willem J. G. Melchers^11^, Heiman F. L. Wertheim^11^, Remco Dijkman^12^, Manon M.C. Holstege^12^, Cornelis J. Vermeulen^12^, Sander Schuurman^12^, Karin van Leeuwen^13^, Nadia Keijzer^13^, Lianne Koets^13^, and Marco Koppelman^13^

RIVM COVID-19 Molecular epidemiology group:

Lynn Aarts^1^, Jeroen Alblas^1^, Birgit van Benthem^1^, Sanne Bos^1^, Annemarie van den Brandt^1^, Sharon van den Brink^1^, Jeroen Cremer^1^, Timor Faber^1^, Kim Freriks^1^, Rolina van Gaalen^1^, Brechje de Gier^1^, Eveline Geubbels^1^, Janneke van Heereveld^1^, Karim Hajji^1^, Susan van den Hof^1^, Agnetha Hofhuis^1^, Senna van Iersel^1^, Ryanne Jaarsma^1^, Jan van de Kassteele^1^, Annelies Kroneman^1^, Maarten Mulder^1^, Priscila de Oliveira Bressane Lima^1^, Jan Polman^1^, Maarten Schipper^1^, Euníce Then^1^, Bas van der Veer^1^, Ivo van Walle^1^, Sara Wijburg^1^, and Lisa Wijsman^1^

Affiliation 1 can be found on the first page of the paper.

^2^Department of Medical Microbiology, Amsterdam University Medical Center, Amsterdam, the Netherlands.

^3^Department of Viroscience, Erasmus MC, Rotterdam, the Netherlands

^4^Laboratory for Medical Microbiology and Public Health, Labmicta, Hengelo, the Netherlands

^5^Department of Medical Microbiology, Leiden University Medical Center, Leiden, the Netherlands

^6^Medical Microbiology Laboratory, Maasstad Hospital, Rotterdam, the Netherlands

^7^Microvida Laboratory for Microbiology, Elisabeth-TweeSteden Hospital, Tilburg, the Netherlands

^8^Department of Medical Microbiology, Maastricht University Medical Centre (MUMC+), Maastricht, the Netherlands

^9^Department of Medical Microbiology, University Medical Center Groningen, Groningen, the Netherlands

^10^Department of Medical Microbiology, University Medical Center Utrecht, Utrecht, the Netherlands

^11^Department of Medical Microbiology, Radboud University Medical Center, Nijmegen, the Netherlands

^12^Royal GD, Deventer, the Netherlands

^13^Sanquin Diagnostics, Amsterdam, the Netherlands
