# Supplementary material for: Elevated risk of infection with SARS-CoV-2 Beta, Gamma, and Delta variant compared to Alpha variant in vaccinated individuals
Source: Sci Transl Med. 2022 Jul 21:eabn4338. doi: 10.1126/scitranslmed.abn4338 (PMC9580257; doi:10.1126/scitranslmed.abn4338)
Supplement: Supplementary file 1 — Figs. S1 to S3 [file scitranslmed.abn4338_sm.pdf]

Science Translational Medicine
AAAS

Supplementary Materials for

Elevated risk of infection with SARS-CoV-2 Beta, Gamma, and Delta variant compared to Alpha variant in vaccinated individuals

Stijn P. Andeweg *et al.*

Corresponding author: Mirjam J. Knol, mirjam.knol@rivm.nl; Dirk Eggink, dirk.eggink@rivm.nl

DOI: 10.1126/scitranslmed.abn4338

The PDF file includes:

Figs. S1 to S3

Other Supplementary Material for this manuscript includes the following:

MDAR Reproducibility Checklist
Table S1
Data file S1

Supplementary information

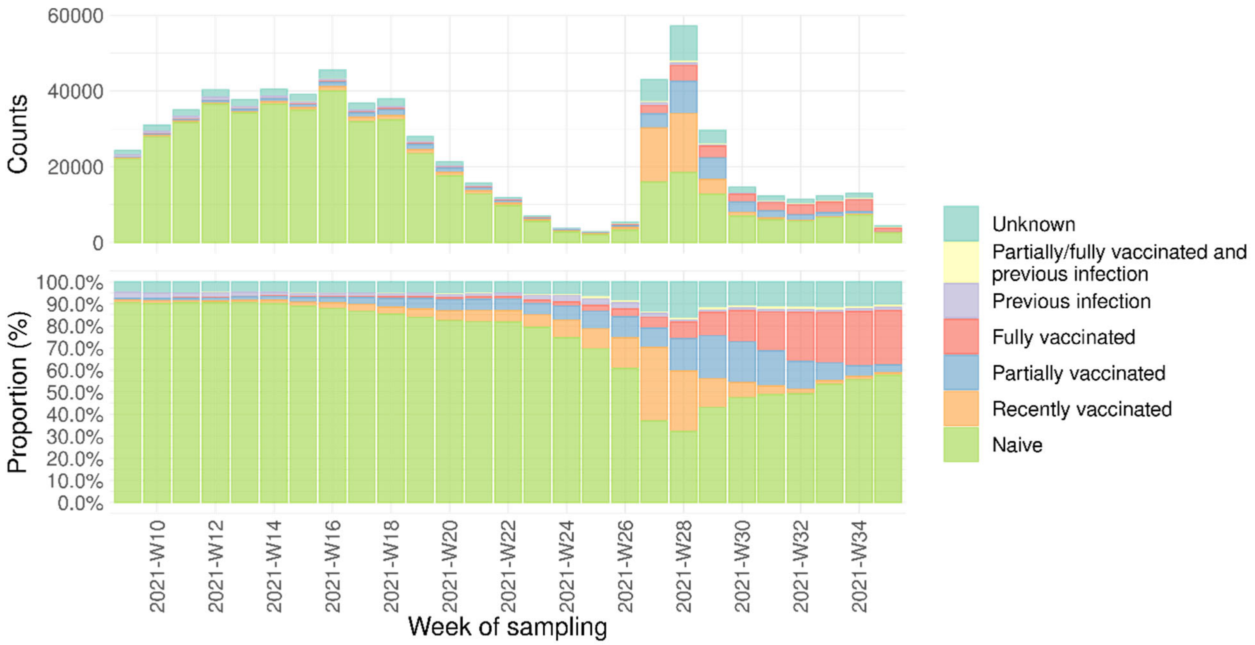

Fig. S1 Immune status of notified SARS-CoV-2 cases in the Netherlands. *Number of naïve (unvaccinated and no known previous infection), recently vaccinated, partially vaccinated, fully vaccinated, reinfected, partially/fully vaccinated and reinfected, and unknown documented SARS-CoV-2 positive individuals from March 1 to August 31, 2021 (upper panel) and proportion of the respective groups (lower panel) per week of sampling (in ISO 8601 format).*

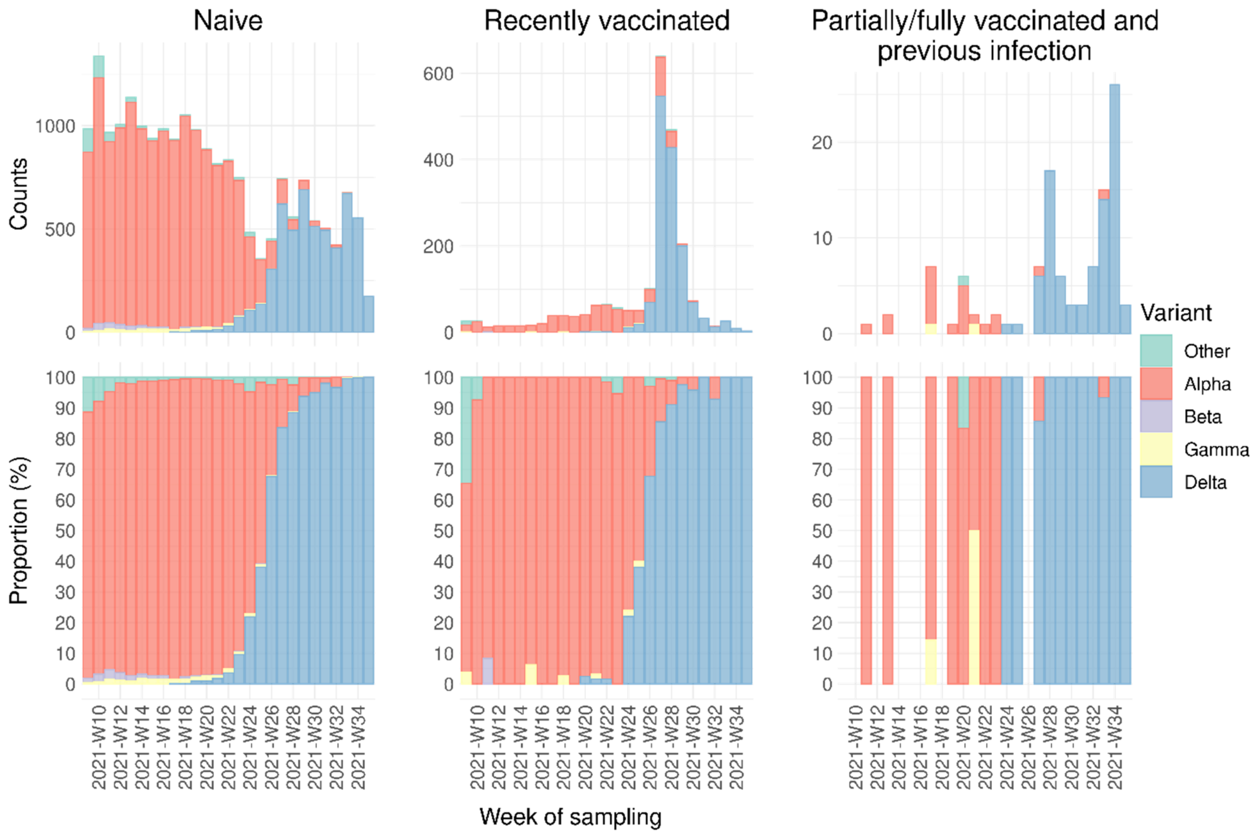

Fig. S2 Variants found in SARS-CoV-2 positive samples of individuals with naïve (unvaccinated and no known previous infection), recently vaccinated, or vaccine-induced and infection-induced immune status. *Number of naïve, recently vaccinated, partially/fully vaccinated and reinfected documented SARS-CoV-2 positive individuals by variant from March 1 to August 31, 2021 (upper panel) and proportion of the respective groups (lower panel), per week of sampling (in ISO 8601 format) .*

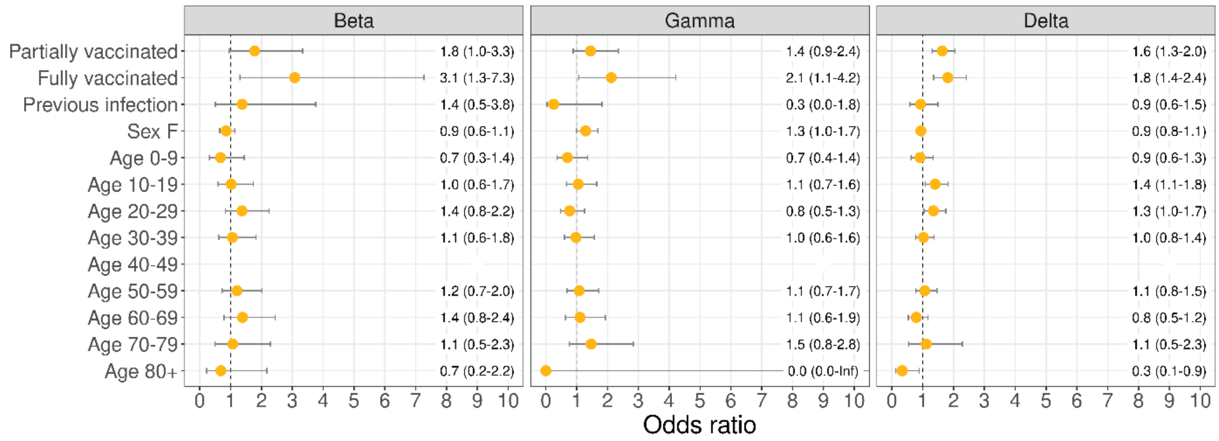

Fig. S3 Odds ratios and 95% confidence intervals for the full model. *Displaying the association between immune status, 10-year age group (40-49 is reference) and sex and the Beta, Gamma or Delta variant with adjustment for week of sampling.*
