# Supplementary material for: Elevated risk of infection with SARS-CoV-2 Beta, Gamma, and Delta variant compared to Alpha variant in vaccinated individuals
Source: Sci Transl Med. 2022 Jul 21:eabn4338. doi: 10.1126/scitranslmed.abn4338 (PMC9580257; doi:10.1126/scitranslmed.abn4338)
Supplement: Supplementary file 2 — MDAR Reproducibility Checklist [file scitranslmed.abn4338_reproducibility_checklist.pdf]

Materials Design Analysis Reporting (MDAR)
Checklist for Authors

The MDAR framework establishes a minimum set of requirements in transparent reporting applicable to studies in the life sciences (see Statement of Task: doi:10.31222/osf.io/9sm4x.). The MDAR checklist is a tool for authors, editors, and others seeking to adopt the MDAR framework for transparent reporting in manuscripts and other outputs. Please refer to the MDAR Elaboration Document for additional context for the MDAR framework.

For all that apply, please note where in the manuscript the required information is provided.

Materials:

Newly created materials	indicate where provided: page no/section/legend)	n/a
The manuscript includes a dedicated "materials availability statement" providing transparent disclosure about availability of newly created materials including details on how materials can be accessed and describing any restrictions on access.		X

Antibodies	indicate where provided: page no/section/legend)	n/a
For commercial reagents, provide supplier name, catalogue number and RRID, if available.		X

DNA and RNA sequences	indicate where provided: page no/section/legend)	n/a
**Short novel DNA or RNA including primers, probes:** Sequences should be included or deposited in a public repository.	Sequences are deposited on GISAID and the list of accession numbers is provided in Table S1.	

Cell materials	indicate where provided: page no/section/legend	n/a
**Cell lines:** Provide species information, strain. Provide accession number in repository **OR** supplier name, catalog number, clone number, **OR** RRID.		X
**Primary cultures:** Provide species, strain, sex of origin, genetic modification status.		X

Experimental animals	indicate where provided: page no/section/legend)	n/a
**Laboratory animals or Model organisms:** Provide species, strain, sex, age, genetic modification status. Provide accession number in repository **OR** supplier name, catalog number, clone number, **OR** RRID.		X
**Animal observed in or captured from the field:** Provide species, sex, and age where possible.		X

Plants and microbes	indicate where provided: page no/section/legend)	n/a
**Plants:** provide species and strain, ecotype and cultivar where relevant, unique accession number if available, and source (including location for collected wild specimens).		X
**Microbes:** provide species and strain, unique accession number if available, and source.		X

Human research participants	indicate where provided: page no/section/legend) or state if these demographics were not collected	n/a
If collected and within the bounds of privacy constraints report on age, sex and gender or ethnicity for all study participants.	Aggregated data on age and sex is provided in Table 1. The gender and ethnicity of the study participants is unknown.	

Design:

Study protocol	indicate where provided: page no/section/legend)	n/a
If study protocol has been pre-registered, provide DOI. For clinical trials, provide the trial registration number **OR** cite DOI.	Study protocol is not publicly registered, only within the Dutch National Institute for Public Health and the Environment (RIVM).	

Laboratory protocol	indicate where provided: page no/section/legend)	n/a
Provide DOI **OR** other citation details if detailed step-by-step protocols are available.	A step-by-step protocol is available and referred to in reference 38. DOI: [illegible]	

Experimental study design (statistics details)		
**For in vivo studies:** State whether and how the following have been done	**indicate where provided: page no/section/legend. If it could have been done, but was not, write not done**	**n/a**
Sample size determination		X
Randomisation		X
Blinding		X
Inclusion/exclusion criteria	The criteria are provided in the Materials and Methods, in the 'Study design' and 'Data' sections (pages 10-11).	

Sample definition and in-laboratory replication	indicate where provided: page no/section/legend	n/a
State number of times the experiment was replicated in laboratory.		X
Define whether data describe technical or biological replicates.		X

Ethics	indicate where provided: page no/section/legend	n/a
**Studies involving human participants:** State details of authority granting ethics approval (IRB or equivalent committee(s), provide reference number for approval.	The Centre for Clinical Expertise at the National Institute for Public Health and the Environment (RIVM) assessed the research proposal following the specific conditions as stated in the law for medical research involving human subjects. The work described was exempted for further approval by the ethical research committee. This is described in the 'Ethics' section (page 11-12).	
**Studies involving experimental animals:** State details of authority granting ethics approval (IRB or equivalent committee(s), provide reference number for approval.		X
**Studies involving specimen and field samples:** State if relevant permits obtained, provide details of authority approving study; if none were required, explain why.		X

Dual Use Research of Concern (DURC)	indicate where provided: page no/section/legend	n/a
If study is subject to dual use research of concern regulations, state the authority granting approval and reference number for the regulatory approval.		X

Analysis:

**Attrition**	**indicate where provided: page no/section/legend**	**n/a**
Describe whether exclusion criteria were preestablished. Report if sample or data points were omitted from analysis. If yes report if this was due to attrition or intentional exclusion and provide justification.	Exclusion criteria were preestablished. Only data was excluded when information was missing (on vaccination status, testing date, variant typing, etc.).	

**Statistics**	**indicate where provided: page no/section/legend**	**n/a**
Describe statistical tests used and justify choice of tests.	A multinomial logistic regression is used. We made the choice for this type of statistical model as we compare multiple variant (Beta, Gamma, and Delta variants) to one reference level (Alpha variant).	

**Data availability**	**indicate where provided: page no/section/legend**	**n/a**
For newly created and reused datasets, the manuscript includes a data availability statement that provides details for access or notes restrictions on access.	Data used in all figures is provided in Data file 1. WGS data is available on GISAID. Both datasets are described in the Data and materials availability section (page 20).	
If newly created datasets are publicly available, provide accession number in repository **OR** DOI **OR** URL and licensing details where available.	The raw case-based data are protected and are not publicly available due to data privacy laws, because of potential for identifiability of individuals. On request aggregated data is available.	
If reused data is publicly available provide accession number in repository **OR** DOI **OR** URL, **OR** citation.		X

**Code availability**	**indicate where provided: page no/section/legend**	**n/a**
For all newly generated custom computer code/software/mathematical algorithm or re-used code essential for replicating the main findings of the study, the manuscript includes a data availability statement that provides details for access or notes restrictions.	Code is available on GitHub and Zenodo. Access is described in the Code availability statement (page 20).	
If newly generated code is publicly available, provide accession number in repository, **OR** DOI **OR** URL and licensing details where available. State any restrictions on code availability or accessibility.	Newly generated code for statistical analysis, figures, and tables used in this study is publicly available (DOI: doi.org/10.5281/zenodo.6670333).	
If reused code is publicly available provide accession number in repository **OR** DOI **OR** URL, **OR** citation.		X

Reporting

MDAR framework recommends adoption of discipline-specific guidelines, established and endorsed through community initiatives. Journals have their own policy about requiring specific guidelines and recommendations to complement MDAR.

**Adherence to community standards**	**indicate where provided: page no/section/legend**	**n/a**
State if relevant guidelines (e.g., ICMJE, MIBBI, ARRIVE) have been followed, and whether a checklist (e.g., CONSORT, PRISMA, ARRIVE) is provided with the manuscript.		X
